# Indolent Tumour of the Upper Lip: A Case of Lobulated Acinic Cell Carcinoma

**DOI:** 10.7759/cureus.95777

**Published:** 2025-10-30

**Authors:** Soumya Makarla, Radhika Bavle, Manish K Singh, Swathi Singh, Satyajit Topajiche

**Affiliations:** 1 Oral and Maxillofacial Pathology, Care Oral Pathology Services, Bengaluru, IND; 2 Department of Oral and Maxillofacial Pathology, Krishnadevaraya College of Dental Sciences, Bengaluru, IND; 3 Department of Oral and Maxillofacial Pathology, Krishnadevaraya College of Dental Sciences and Hospital, Bengaluru, IND; 4 Oral and Maxillofacial Surgery, Manaswa Facial Surgery and Impant Centre, Bengaluru, IND; 5 Oral and Maxillofacial Surgery, Manaswa Facial Surgery and Dental Implant Center, Bengaluru, IND; 6 Oral and Maxillofacial Pathology, KLE Society’s Institute of Dental Sciences, Bengaluru, IND

**Keywords:** acinic cell carcinoma, dog-1, ki-67, lobular pattern, low grade, periodic acid–schiff (pas), prognostic markers, solid variant, upper lip

## Abstract

A majority of minor salivary gland neoplasms are encountered more frequently on the upper lip when compared to lower lip lesions. Although upper lip tumours generally turn out to be benign adenomas in the form of monomorphic adenomas and pleomorphic adenomas in 90% of the cases, rarely, a few indolent masses turn out to be carcinomas. Here, we report a long standing indolent upper lip swelling, which turned out to be a low-grade acinic cell carcinoma (ACiCa). Minor salivary gland tumours (MSGTs), considered as benign clinically, should be evaluated histopathologically for the confirmation of diagnosis, especially to rule out malignancy. Detailed, systematic histopathological and immunohistochemical (IHC) assessment for validation and confirmatory diagnosis is imperative. An added evaluation for tumour outcome with affirmative markers (DOG1), proliferative markers (Ki67), add value in diagnosis and prognostication of this tumour.

## Introduction

Swellings of the upper lip and labial mucosa are home to many types of pathologies. They can be reactive lesions, traumatic, foreign body granulomas, granulomatous disorders, or tumours [1]. A single lump in the upper lip narrows down the diagnosis to either a reactive, traumatic, or neoplastic lesion. A well-circumcised lesion is indicative of a long-standing, benign tumour of the salivary gland, associated with the minor salivary glands [1].

Indolent swellings generally are adenomas of the salivary gland, with pleomorphic adenomas being more common and monomorphic adenomas rarer [2]. Clinically presenting as persistent but asymptomatic lumps or swellings for long periods of time, these tumours can be challenging. Their evaluation is important as some of them can be low-grade carcinomas in the guise of adenomas.

In terms of location, minor salivary gland tumours (MSGTs) most commonly occur in the palate, followed by lip, buccal mucosa, tongue, and floor of the mouth [3,4]. Salivary gland neoplasms of the lip are encountered more frequently on the upper lip (84-85%) as compared to the lower lip (15-16%) [2].

A change in the clinical picture is one of the clues for conversion into carcinoma. Upper lip MSGTs account for 67% of adenomas and 33% of carcinomas in the oral cavity [4].

Low-grade carcinomas of the lip are primarily mucoepidermoid carcinoma, polymorphous adenocarcinoma, and adenoid cystic carcinoma [3,5].

Here, we report a case of a small mass on the right upper lip of a geriatric female patient, which was diagnosed as a low-grade adenocarcinoma. Grading of the tumour becomes vital as it dictates the treatment protocol in such cases.

## Case presentation

An 82-year-old female presented with a small indolent swelling on the upper lip on the right side of 15 months' duration. It was approximately 1.5 cm in diameter, with clearly defined borders, well palpable, soft to firm, and non-tender. Extra-orally, the swelling was subtle and showed mildly stretched skin on the upper lip, which was normal in colour and consistency. From the labial mucosa, the swelling appeared well-delineated, covered by stretched, normal-appearing mucosa. On palpation, the small lump was neither mobile nor fluctuant. It was completely asymptomatic, but with a history of mild progression in size over the last few months.

A provisional diagnosis of a mucocele or a minor salivary gland adenoma was considered. The lesion was excised with a 5 mm safe border for further histopathological examination. Surgical detailing revealed a well-circumscribed soft to firm mass, which was removed with ease.

**Figure 1:**
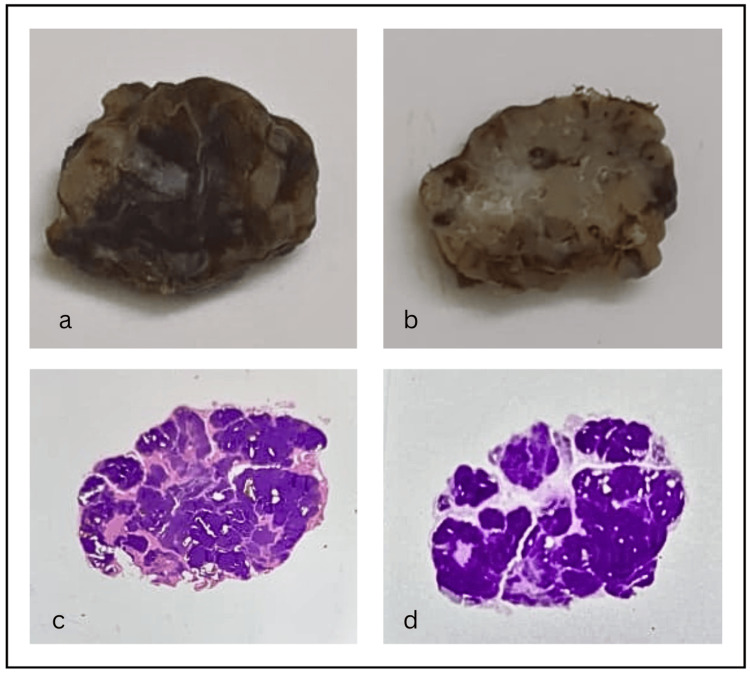
Gross and macroscopic images of the low-grade adenocarcinoma. A: Gross image of the excised lump (1.5 cm) exhibiting a well-circumscribed nodular lesion. B: On cut the section, it is creamish-brown and homogeneous. C: Macroscopic stained section of the lesion, highly basophilic and lobulated in appearance (H&E). D: Deeply stained lesional cells, highly positive for special stain periodic acid-Schiff (PAS).

The grossing showed a very well demarcated 1.5 cm, soft to firm lump, which appeared homogenous on cut section (Fig. 1a, 1b). On closer detailing, it appeared to be lobulated and encapsulated. The macroscopic stained section on the slide appeared overtly basophilic and lobulated (Fig. 1c, 1d). The tumour appeared to be well defined, with a capsular/pseudocapsular structure.

**Figure 2:**
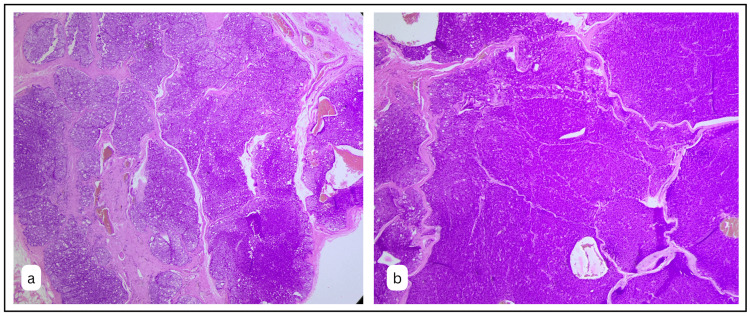
A, B: Scanner view revealing basaloid tumour cells arranged in well-delineated lobes and islands, intervened by cystic cavitations with hemorrhage (H&E x40).

Microscopic analysis of the given section on scanner view disclosed a complete basaloid hyperchromasia of tumour cells. The lesion was well delineated at the periphery, and the tumour cells were in well-formed lobes and islands (Fig. 2a). The tumour cells were closely arranged in the form of islands and follicles, closely packed around each other with intervening mature connective tissue in the form of a few bands of collagen (Fig. 2b).

**Figure 3:**
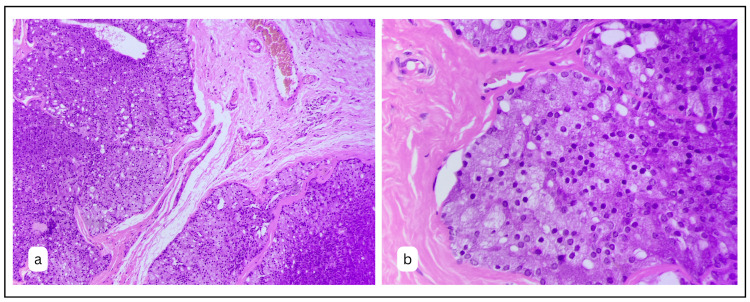
A: The section shows tumour islands and follicles separated by intervening mature fibrous connective tissue (H&E x100). B: Higher magnification exhibiting predominantly ovoid cells with granular cytoplasm and hyperchromatic round monotonous prominent nucleus throughout the lesion (H&E x400).

The tumour cell population was largely made up of oval cells with a round, prominent nucleus of monotonous nature across all cells. Predominantly, the tumour was made up of large oval/polygonal cells studded with basophilic granules (Fig. 3a).

A small population of cells showed vacuolated and clear cells. Some of the tumour islands showed the presence of palisading columnar cells at the periphery of the tumour follicles. On a higher magnification, dense coarse granules were observed within the cells, contributing to the richly cytoplasmic granular cells of the tumour (Fig. 3b).

**Figure 4:**
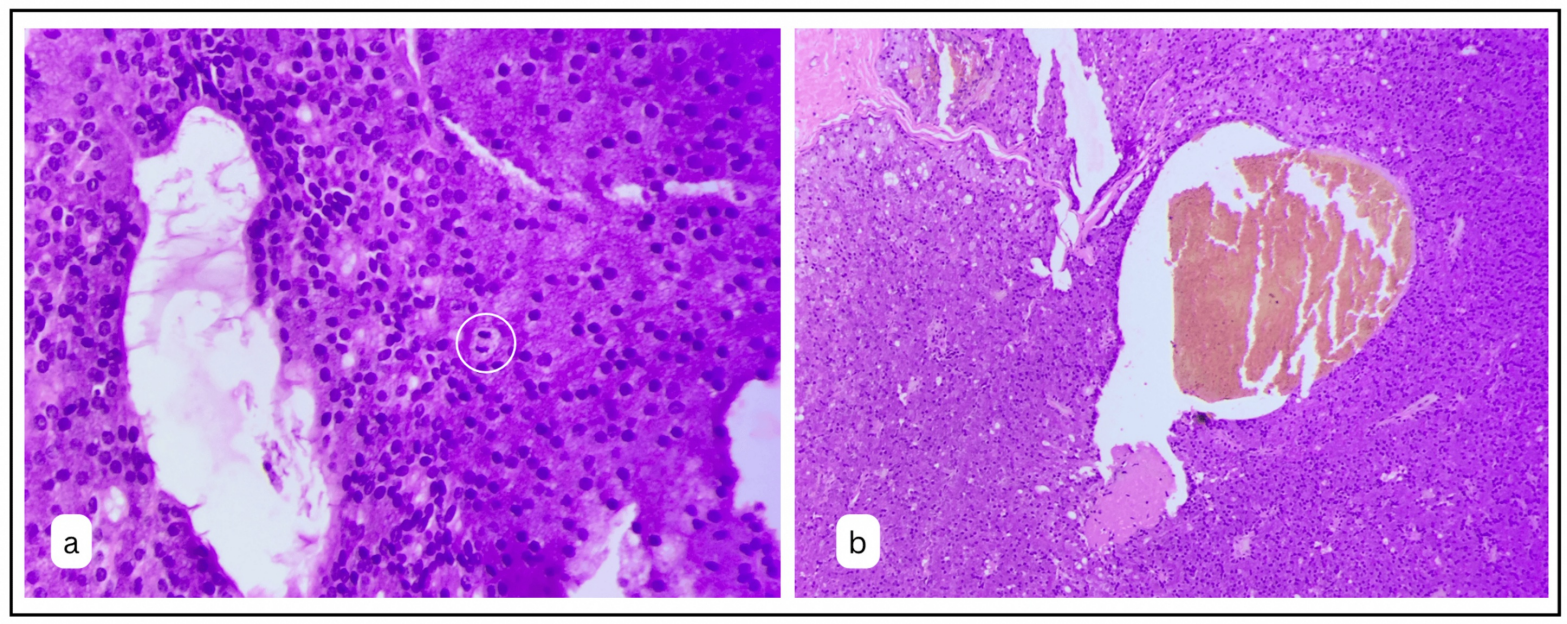
A: Photomicrograph exhibiting monotonous nature of the cells with little pleomorphism and a few mitotic figures (H&E x400). B: Few areas of the tumour showing necrotic foci filled with hemorrhagic fluid (H&E x40).

Pleomorphism of the cell and nucleus was very mild, but a few mitotic figures were noted, 4-5 per 10 HPF (Fig. 4a). One area of the tumour cells showed necrosis surrounded by the dysplastic tumour cells. A few areas in the tumour mass showed cystic areas filled with haemorrhagic fluid. These areas were lined by the tumour cells (Fig. 4b).

Based on the histopathological presentation, the diagnosis of acinic cell carcinoma (ACiCa) was considered. The arrangement was similar to the solid variant of ACiCa, also frequently called a lobulated pattern. 

The relevant differential diagnoses, clinically and largely histopathological, were considered and ruled out based on the histopathological evaluation of pattern and cell types, special stains, and immunohistochemical (IHC) analysis.

**Figure 5:**
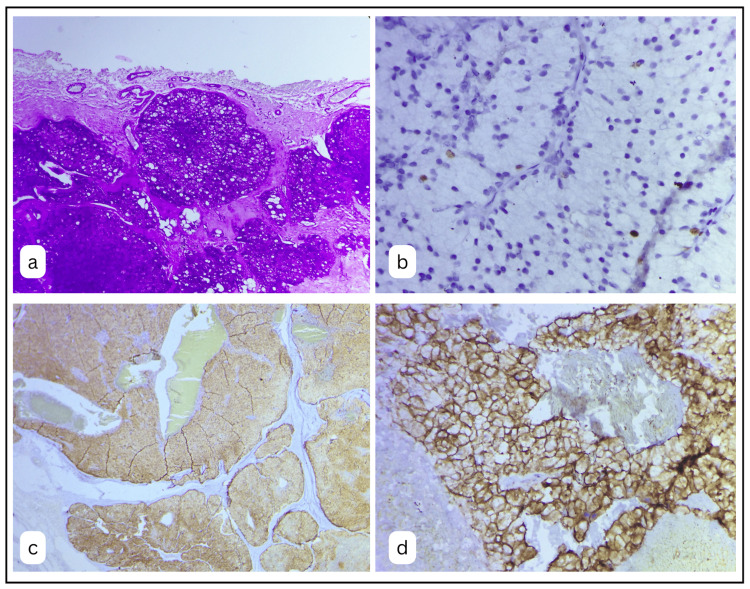
Special stains and Immunohistochemical markers employed for the confirmatory diagnosis of acinic cell carcinoma. A: PAS-stained section with zymogen granules taking up a deep magenta colour in most of the cells (PAS x100). B: The section shows few of the tumour nuclei positive for Ki67 with an index of 3-5% (IHC – Ki67 x400). C: Photomicrograph exhibiting islands with 99% of tumour cells positive for DOG1 (IHC – DOG1 x40). D: Higher magnification with tumour cells showing cytoplasmic and membranous positivity (IHC – DOG1 x200).

On the first rung of evaluation, periodic acid-Schiff (PAS) staining was undertaken, which showed a dense collection of deep magenta, zymogen granules in almost all the tumour cells. The positively stained granules were well packed in the cytoplasm of the tumour cells, which made up the largest population of cell types in the tumour (Fig. 5a). A small segment of clear cells did not show the presence of PAS staining. 

A further detailed IHC evaluation was done for confirmation and prognosis. To assess the proliferation index, Ki67 was used. Strong nuclear positivity was noted in a few tumour cells, 3-5%, patchy in presentation (Fig. 5b).

DOG-1 and p63 were done for an affirmation of the diagnosis. Moreover, 99% of tumour cells were positive for DOG-1, showing dense cytoplasmic and membranous staining (Fig. 5c, 5d). p63 was completely negative in almost all the tumour cells. 

Histopathological prognostication was considered on the basis of multiple factors. It was graded as low grade, based on tumour size, gender, no evidence of metastatic distribution, no evidence of marked pleomorphism, low Ki-67 index, and no evidence of extra capsular invasion/extension of the tumour mass.

The special stain and IHC confirmed the histopathological diagnosis. A diagnosis of acinic cell carcinoma (low-grade, solid variant) was given. Hence, a surgical excision with a 0.5 cm safe margin was considered ample for the treatment, along with follow-up and evaluation for a minimum of eight to 10 years. The patient is healing well with no evidence of any signs of recurrence after one year of follow-up. 

## Discussion

The present case describes a case of ACiCa in an 82-year-old female, which corroborates the global incidence of ACiCas arising more commonly in females and in those over 50 years of age [6,7].

ACiCas are infrequent malignant salivary gland tumours comprising only 6-11% of all salivary gland tumours [6,8]. This tumour tends to affect the parotid gland most frequently, followed by the other major salivary glands and then the minor salivary glands [6,8].

Among the minor salivary gland tumours, those presenting on the lip account for 13-17% of salivary gland tumours [7,9], the commonest site being the palate (55-78%), followed by buccal mucosa and the retromolar area [4,5]. In contrast to this, ACiCas have been found to mainly occur in the buccal mucosa (33%) and upper lip (13-17%) [8].

The upper lip is a frequent site for adenomas, considering mucoceles to be more prevalent on the lower lip [1,9]. The common salivary gland tumours to be diagnosed on the upper lip are benign adenomas in the form of pleomorphic adenomas, canalicular and basal cell adenomas [3]. About 55-59% of cases might simulate an adenoma, which, on histopathological evaluation, are finally diagnosed as indolent or low-grade carcinomas [3,5].

Swellings of the upper lip are detected quite early because of the location, functionality, and aesthetics. Generally, the mass does not grow beyond a few centimetres [1]. They present as a well-demarcated, soft to firm mass with a normal mucosal covering. They might show crater or ulcer formation in rare instances [6,8]. Similarly, in the present case, the growth was detected early with a dimension of 1.5 cm on the upper right labial mucosa. Although asymptomatic, the case had a mild progressive history of enlargement. 

Based on the location and size, on provisional diagnosis, these tumours are treated by excisional biopsy. In the present case, a long-standing, asymptomatic lump on the upper lip was treated surgically with a safe, free margin. 

The grossing showed a very well-delineated cellular mass, firm to soft in consistency, with a lobulated appearance on the cut section. A similar gross presentation has been described by various authors [8,10]. A unique macroscopic evaluation disclosed a predominantly lobulated, cellular tumour of basophilic cells (Fig. 1).

ACiCas are characterised by tumour cells exhibiting serous acinar differentiation owing to the presence of hematoxyphilic secretory granules in the cytoplasm of the cells [5,9]. These tumours are learnt better with their variants - solid, microcystic, papillary cystic, and follicular. The cells that characterise this tumour are acinar, intercalated, ductlike, and vacuolated types. The rarer varieties include clear cell and oncocytic changes [8,11].

The solid variant comprises sheets of cells organised into trabeculae or acinar architecture by connective tissue septa. Small lumina interspersed between the aggregates of tumour cells give it a microcystic appearance. Microcystic and papillary cystic types exhibit a higher population of vacuolated cells, although acinar cells predominate in the microcystic variant.

The presence of proliferating glandular epithelium projecting into cystic structures is characteristic of the papillary cystic growth pattern. The size of the cysts might vary; some present with stalks, fronds, or even masses of epithelial cells within the lumina. Intercalated duct-like cells and non-specific glandular cells are more numerous in this type [12].

Follicular pattern is rarely encountered, wherein multiple variably sized round to ovoid cystic spaces lined by columnar or cuboidal cells, containing amorphous eosinophilic material resembling colloids, are evident. The intercystic spaces contain mostly non-specific glandular cells with few vacuolated and acinar cells [12]. The solid variant is termed the 'classic pattern', although the microcystic pattern is more commonly observed. The papillary cystic and follicular variants occur less frequently than the solid pattern [8,9].

On histopathological evaluation, the present case showed a tumour with a well-delineated periphery, predominantly basophilic and cellular in a lobular or organoid pattern. The tumour was divided into lobes of largely prominent cells by hyalinised collagenous fibres. The pattern looked cellular and organised, resembling an organoid sheet pattern. A small population of vacuolated, clear, and cuboidal cells was also seen. An area of follicular pattern was also noted. Foci of necrosis and 3-5 mitosis per 10 HPF were also evident among the tumour cells, which allowed the consideration of a solid variant of ACiCa as the diagnosis. 

Predominantly made up of oval or polygonal cells, with good cytoplasmic body, a prominent round hyperchromatic nucleus and basophilic granules, giving the cells a typical ‘blue dot’ configuration, was observed throughout the tumour tissue. Such a cellular configuration was described by Nasse in the first reported case of ACiCas in 1892 [6]. The other cells noted were vacuolated, clear, and cuboidal cells, accounting for 10-15% of the tumour cells, drawing a histopathological conclusion of ACiCas. 

These tumours tend to undergo a process of dedifferentiation or 'high-grade transformation', wherein areas of high-grade or undifferentiated adenocarcinoma are found with classical ACiCas [8,13]. No such areas could be identified in the current case. 

A well-analysed histopathological profile can, in most instances, confirm a diagnosis of ACiCa using an H&E-stained slide. However, in some cases, owing to different variants and variations, it needs to be confirmed by using further IHC evaluation and/or molecular diagnostics.

At this stage, ruling out the differential diagnosis of salivary gland tumours with predominance of basophilic cells was considered. Basal cell adenomas and adenocarcinomas also bear a lobular pattern with basophilic cells but largely contain a clear, small cytoplasmic body. The presence of large oval/polygonal cells with rich cytoplasmic areas studded with basophilic granules excluded the basal cell tumours of salivary glands. Adenoid cystic carcinoma needs to be ruled out in such instances. The absence of classical patterns like cribriform and tubular, no evidence of perineural invasion, and the presence of a capsular element also helped in ruling out adenoid cystic carcinoma.

A largely granular cell population allows consideration of oncocytic change. A densely basophilic presence rules out the eosinophilic granular content of an oncocyte. Mucoepidermoid carcinoma and secretory carcinoma were ruled out on the basis of cell population and pattern analysis. 

A characteristic feature of ACiCa is the presence of cytoplasmic granules (zymogen granules) that are PAS (diastase-resistant)-positive and mucicarmine stain-negative [9]. The tumour cells in the present case exhibited a dense staining of the tumour cells for PAS. 

The tumour cells in ACiCas are very sensitive to markers like DOG-1, EMA, SOX10, and NR4A3, a novel specific marker, and are stained well. They are, on the other hand, negative, largely to markers like p63, S100, p40, mammoglobin, and CK14 [13,14]. The above-reported case was densely ‘positive’ for DOG-1 in almost all tumour cells. 

DOG-1 is a transmembrane ionic channel mediating calcium-dependent chloride secretion in glandular and squamous epithelia. Studies have found that DOG-1 expression is crucial to ascertain the acinar nature of salivary gland tumours. It has been found to highlight an amplified serous acinar histo-type in ACiCas, confirming the diagnosis [15].

The cytoplasmic and membranous positivity of DOG-1 observed in this case was in corroboration with studies by Chênevert et al. and a meta-analysis by Fiorentino V et al. [15,16]. Fiorentino V et al. in their systematic review also observed that DOG1 expression was predominantly seen in well-differentiated ACiCas and thus could be an indicator of lower aggressiveness in such tumours [15].

p63 was negative in the present case, in accordance with the study by Khurram SA and Speight PM. The study concluded that 87.5% of cases were negative for p63, further confirming that ACiCas are negative for myoepithelial markers [17].

Ki-67 is a promising predictor of the proliferation of tumour cells. A proliferative index above 5-10% is considered to be unfavourable [10]. In our case, it was confined to 3-5%, suggestive of a good outcome as a low-grade tumour. 

The other prognostically relevant markers for the evaluation of ACiCas are age (>45), gender (M), size of tumour (>3 cm), and the presence of metastasis clinically [10,18]. Although a definite WHO grading system for ACiCa is yet to be defined, some authors/studies suggest grading this tumour as low grade or high grade based on its proliferative characteristics, such as high mitotic activity (>2 mitoses/10 high-power field), presence of necrosis and pleomorphic cells, tumour extracapsular extension, and positive resection margins [6].

On histopathological evaluation, tumour necrosis, nuclear pleomorphism, increased mitosis, and high Ki-67 index (>5%), presence of dedifferentiated areas, vascular or neural invasion, and extracapsular extension of tumour mass, and depletion of stromal lymphocytic infiltration are deemed high-grade features [11].

In the present case of a female geriatric with a tumour of 1.5 cm without a high proliferation index, absence of marked pleomorphism, no evidence of local or neural invasion, and extracapsular spread justified the grading of low-grade carcinoma. 

Molecular or cytogenetic rearrangement is recurrent (t[4;9] [q13;q31]) in the nuclear receptor subfamily 4, Group A, marker 3 (NR4A3). Wong S et al. and Haller F et al., in their large case series, confirmed a 97-98% specificity for moderate to strong NR4A3 expression in ACiCas. Loss of functional mutation of the CDKN2A/2B gene is also noted in this tumour [19,20].

Since an affirmation was already reached with special stain and IHC, molecular tests were not taken up for the reported case.

The conventional treatment modality for low-grade and lower-stage (I and II) ACiCas is surgical resection with adequate margins. Post-surgical radiotherapy is not suggested for these low-grade tumours, as they exhibit an excellent prognosis of a five-year survival rate of >90% with surgery alone [8]. This was further corroborated by an SEER analysis, which assessed the oncologic benefits of adjuvant radiotherapy [8]. The lip lesion in the present case was accordingly treated by surgical excision with an adequate margin.

## Conclusions

On a concluding note, it is crucial to remove any long-standing or indolent mass, although asymptomatic and not clinically pertinent. A clinical examination or assessment of a mass or lesion showing variation or developing signs and symptoms makes it mandatory to be biopsied. 

Fine-needle aspiration cytology (FNAC) diagnoses of salivary gland tumours should be further confirmed with an incisional or excisional biopsy. A detailed histopathological analysis without sole consideration of only clinical assessment becomes relevant in today’s clinical practice.

A diagnosis of adenocarcinoma of salivary gland tumours entails a detailed histopathological analysis to confirm the criteria for low or high grade lesions, in order to initiate appropriate treatment and prognostication.

Grading for ACiCas goes beyond histopathological findings, necessitating a good clinicopathological correlation to predict the prognosis. In the present case, although a highly cellular and solid pattern was noted, the other parameters for grading steered it into a low grade carcinoma with good prognosis.
